# Supplementary material for: Breast Cancer-Derived Extracellular Vesicle miR-425-5p (miR-425) Promotes Brain Metastasis via Activating Astrocytes Through the Novel miR-425-ZNF24-CCL8 Signaling Axis
Source: Int J Mol Sci. 2026 Mar 31;27(7):3197. doi: 10.3390/ijms27073197 (PMC13073527; doi:10.3390/ijms27073197)
Supplement: Supplementary file 1 [file ijms-27-03197-s001.zip › ijms-4165233-supplementary.pdf]

Supplementary Figures

Supplementary Figure 1

(Related to Figure 2)

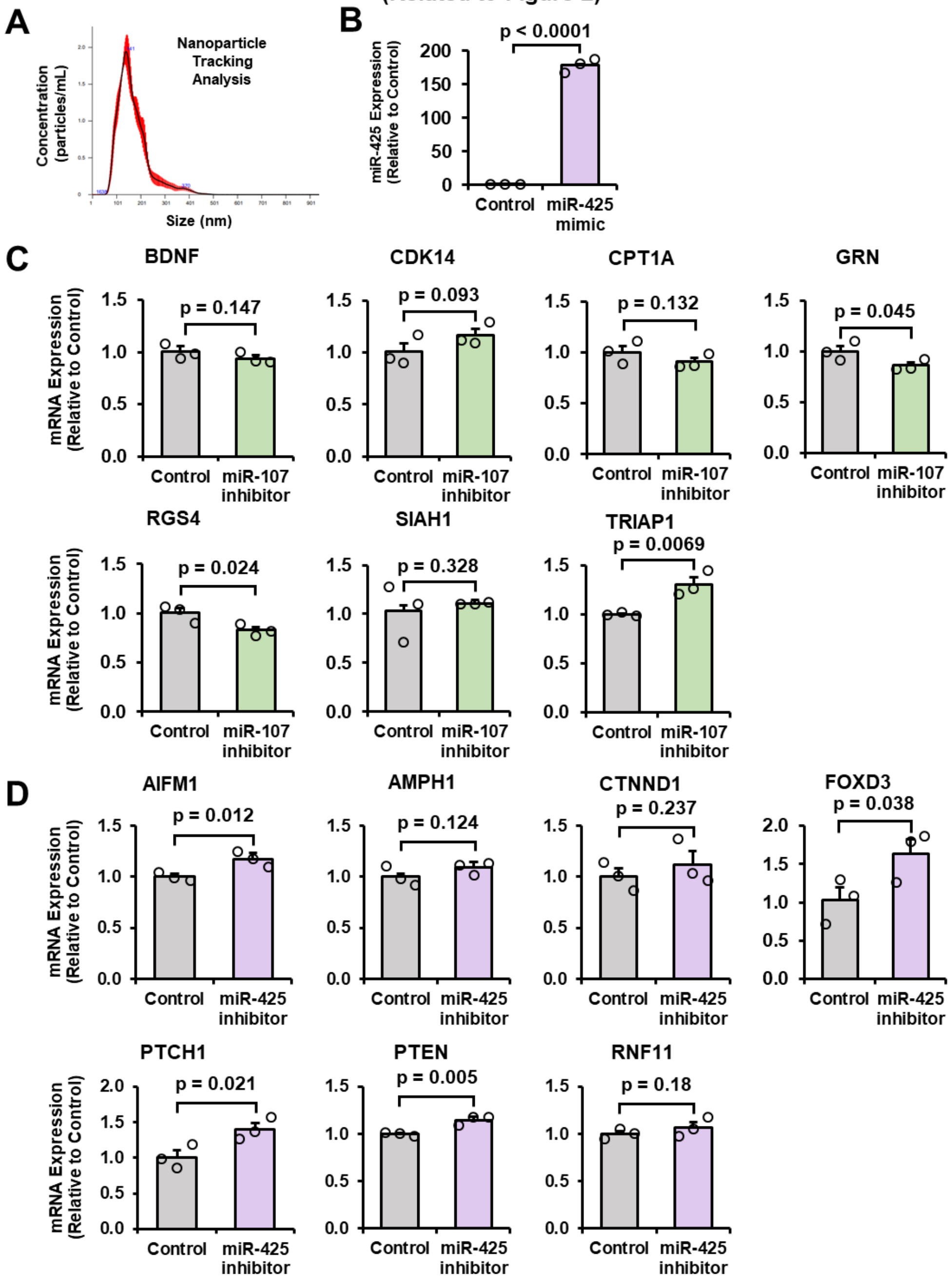

Supplementary Figure S1: Validation of EV isolation, miR-425 overexpression, and miR-107 and miR-425 inhibitors. A) EV isolation via the ExoQuick Kit was validated via Nanoparticle Tracking Analysis (NTA). **B)** Validation of miR-425 overexpression in CN34 cells via miR-RT-qPCR. **C)** miR-107 inhibitor was validated via RT-qPCR of previously published miR-107 target genes. **D)** miR-425 inhibitor was validated via RT-qPCR of previously published

miR-425 target genes. Fold change was calculated in Panels B-D. Student's *t*-test was used in Panels B-D. N = 3 experimental replicates unless otherwise indicated.

Supplementary Figure 2

(Related to Figure 4)

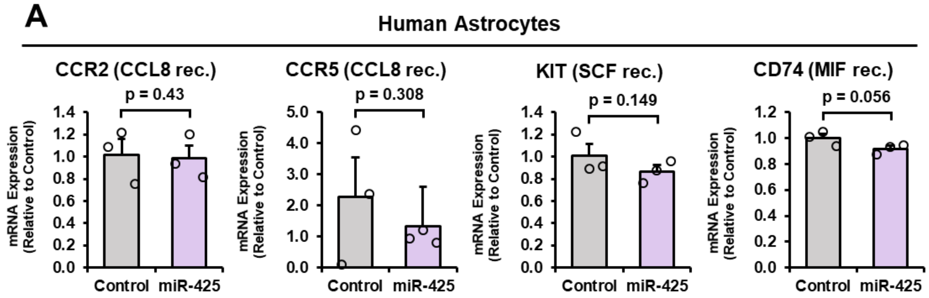

Supplementary Figure S2: CCL8, SCF, and MIF receptor expression in human astrocytes. A) CCR2 (CCL8 receptor), CCR5 (CCL8 receptor), KIT (SCF receptor), and CD74 (MIF receptor) mRNA expression in astrocytes transfected with the miR-425 mimic. mRNA levels measured with RT-qPCR. Fold change was calculated in Panel A. Student's *t*-test was used in Panel A. N = 3 experimental replicates unless otherwise indicated.

Supplementary Figure 3

(Related to Figure 8)

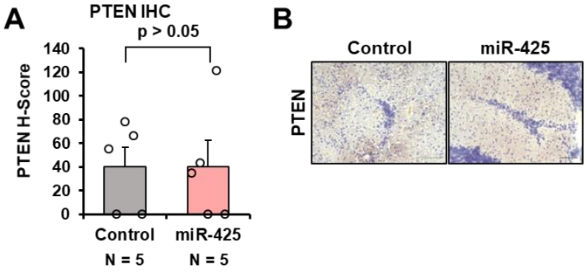

Supplementary Figure S3: PTEN protein expression in brain metastases from mice intracardially injected with breast cancer cells overexpressing miR-425. A) PTEN expression is not significantly different in brain metastases from the control or miR-425-overexpressing groups. **B)** Representative IHC images at 20x magnification. Scale bar indicates 100 µm.

Supplementary Tables

Supplementary Table S1:

RT-qPCR Primer Sequences:

Target Gene	Forward Sequence (5'-3')	Reverse Sequence (5'-3')
AIFM1	TTGAGAATGGTGGTGTGGCT	AGACTTCTTGGAGTACCTCCTGT
AMPH1	CGAGAACTCCGAGGATATTTAGC	CCCATACCAGTCAGGCTCAT
BCOR	CGCTCCTCGCTGAACGC	CGCCATGTTGACGGTTCGC
BDNF	AGATCTTGGGGGAAACACTGC	TAGGGCTTTCTTTCACCGGG
CCL8	TGTCCCAAGGAAGCTGTGAT	TGGAATCCCTGACCCATCTCT
CDK14	GATGTGTGACCTCATTGAGCC	CAATGCGACTGAAACTCTCCG
CPT1A	TGTCCAGCCAGACGAAGAAC	ATCTTGCCGTGCTCAGTGAA
CTNND1	ATGAGTGGTTCTCCAGAGGGA	GCAGAGCAGAGCGGATGTAT
CREB1	GTGACGGAGGAGCTTGTACC	GGACTTGAACTGTCTGCCCA
FOXD3	GCAACTACTGGACCCTGGAC	CTGTAAGCGCCGAAGCTCT
GAPDH	ACTGCCAACGTGTCAGTGG	GTGTCGCTGTTGAAGTCAGA
GFAP	CTGCTGCCTTTAGTCGCTGA	CTGCGGGTGGAATTTGGTGA
GRN	ATCTTTACCGTCTCAGGGACTT	CCATCGACCATAACACAGCAC
KITLG	AGCCAGCTCCCTTAGGAATGA	TGCCCTTGTAAGACTTGGCTG
MIF	ATCGTAAACACCAACGTGCC	TGCTGTAGGAGCGGTTCTG
PTCH1	GGGTGGCACAGTCAAGAACAG	TACCCCTTGAAGTGCTCGTACA
PTEN	AACTTGCAATCCTCAGTTTG	CTACTTTGATATCACCACACAC
RGS4	AGAGGAAAGGCATTGGGAGTC	GCTAAGCCTGTAGGGGTCTC
RNF11	TCTCCCTGCTTCACGAGTCT	AGTCTGGCTAGGTGTTGGGT
SIAH	AGGACCCTACCCAGTGAATCT	GACCCAAATTCGCGTCTGAG
TRIAP1	ATTGCCAGCTCTCAACCCAA	AAGGCAAATGAAGCGAGCAC
VEGFA	TGTCTAATGCCCTGGAGCCT	TTAACTCAAGCTGCCTCGCC
ZNF24	GGAGGTTTGCGCCGGAGT	ACAGACGGCAAAGTTCTCGG
